# Craniosynostosis

**Published:** 2012-03-10

**Authors:** Andrew W. Hoey, Benjamin S. Carson, Amir H. Dorafshar

**Affiliations:** ^a^King's College School of Medicine, London, UK; ^b^Johns Hopkins Medical Institute, Baltimore, Md.

## DESCRIPTION

An 8-month-old baby boy presents with trigonocephaly and undergoes anterior cranial vault reconstruction for metopic craniosynostosis.

## QUESTIONS

1. **What are the types of craniosynostosis?**
2. **What is the incidence of craniosynostosis?**
3. **What are the goals of craniosynostosis surgery?**
4. **If left untreated, what complications are associated with craniosynostosis?**
5. **What is the usual operative sequence for the correction of this disorder?**

## DISCUSSION

Craniosynostosis is a congenital defect in which one or more of the cranial sutures close prematurely, therefore changing the growth pattern of the skull. Worldwide, it affects 1 in 2000 to 2500 live births each year.^15^ Despite being a known condition for centuries, modern surgical management has only developed over the past 100 years.^7^ Success of surgery is dependent on the recognition and understanding of the growth of the skull during infancy and childhood.

Craniosynostosis is classified according to suture involvement, according to associated malformation (in syndromic cases), and increasingly by genetic anomalies.^4^

Fusion of the sagittal suture, scaphocephaly, is the most common type of craniosynostosis, accounting for between 40% and 55% of nonsyndromic cases.^2^ The closure of this suture results in the head growing longer and narrow, instead of wide. Scaphocephaly is more common in boys than in girls and those affected tend to have a broader forehead. The next most common form is anterior plagiocephaly. Involving the coronal suture, it affects girls more often than boys and accounts for 20% to 25% of nonsyndromic cases.^2^ Closure of both coronal sutures is known as brachycephaly and results in the child's head expanding laterally.^15^ Another form is trigonocephaly, the reported incidence of which is highly variable, ranging from 5% to 50% of all cases, with an average of approximately 10%.^14^ It results from the fusion of the metopic sutures, which was seen in the case discussed earlier. The rarest form is posterior plagiocephaly, and it results from the fusion of the lamboid suture and accounts for 2% to 4% of nonsyndromic cases.^2^^,^^6^ In addition, 5% to 15% of cases involve more than suture.^2^ Closure of all of the cranial sutures is known as pansynostosis, which presents with a characteristic Kleeblattschädel or cloverleaf skull.

Recent studies have shown an increase in the number of patients being diagnosed with craniosynostosis, with a relative increase in the proportion of patients being diagnosed with trigonocephaly.^4^ However, the number of patients who underwent surgery remained unchanged. This may reflect an increase in the recognition and diagnosis of less severe forms of craniosynostosis.^8^

Despite a strong genetic link being identified with craniosynostosis, most cases arise in families with no past history of the condition. However, a substantial amount, from 15% to 40% are associated with recognized syndromes, including Apert, Carpenter, Pfeiffer, Crouzon, and Chotzen syndromes.^2^

The premature closure of cranial sutures results in the skull failing to grow at an appropriate rate to match that of the brain. As a result, the growing brain puts additional pressure on the surrounding malleable neonatal calvarium, resulting in an associated cranial deformity. Therefore, the primary goal of surgery is to restore the normal shape of the skull. This is achieved, as discussed earlier, by the removal of the prematurely fused sutures and reconstruction of the affected parts of the skull, which results in an aesthetic improvement to the appearance of the child's head.

Failure to treat craniosynostosis can lead to significant deformity of the head, which can become permanent if left uncorrected.^11^ As the brain continues to grow against a non-expanding skull, the abnormality in the growth of the brain can occasionally result in seizures, developmental delay and may compromise visual function.^13^ This pattern of findings is more typical in patients with multiple suture craniosynostosis related to increased intracranial pressures; however, neuropsychololgical deficits have been reported in studies of patients with single suture craniosynostosis.^1^ The mechanisms of this are unclear, given the fact that the open sutures should be able to compensate to prevent elevated intracranial pressures; however, some authors have attributed this to neuroanatomical differences found in these patients.^10^ There is much controversy in the literature regarding the optimal timing of surgical intervention in patients with craniosynostosis. Although debatable, operating earlier can theoretically prevent neurocognitive impairments; however, intervention too early involves too great a risk for life-threatening blood loss and can lead to recurrence of the cranial vault abnormality. Operating later increases the possibility of forming permanent cranial defects^13^ but results in a safer surgery with less risk of relapse. Currently, most surgeons would agree that operating between the 6- and 12-month window as the optimum time period.^7^^,^^9^

The operative procedure for the correction of this disorder is dependent on the type of craniosynostosis, whether it involves a single or multiple sutures and the time of presentation. If the patient presents at less than 3 months of age with a single suture fusion, an endoscopic strip craniectomy and a period of helmet therapy is recommended.^5^^,^^12^ However, if the patient is older than 3 months, or has a multisuture craniosynostosis, then the recommended course is an open calvarial vault reconstruction at 9 to 12 months of age. Delaying large craniofacial procedures such as these results in significantly lower patient morbidity and mortality.^3^ Lifelong postoperative follow-up is recommended to allow for any major or minor revisions to be considered as the child develops.

## OPERATIVE PROCEDURE

A zig-zag stealth scalp incision was made and an anterior scalp flap was advanced along the subgaleal plane to the level of 1 cm above the supraorbital rims. The fronto-orbital regions were then dissected out.

**Figure 2:**
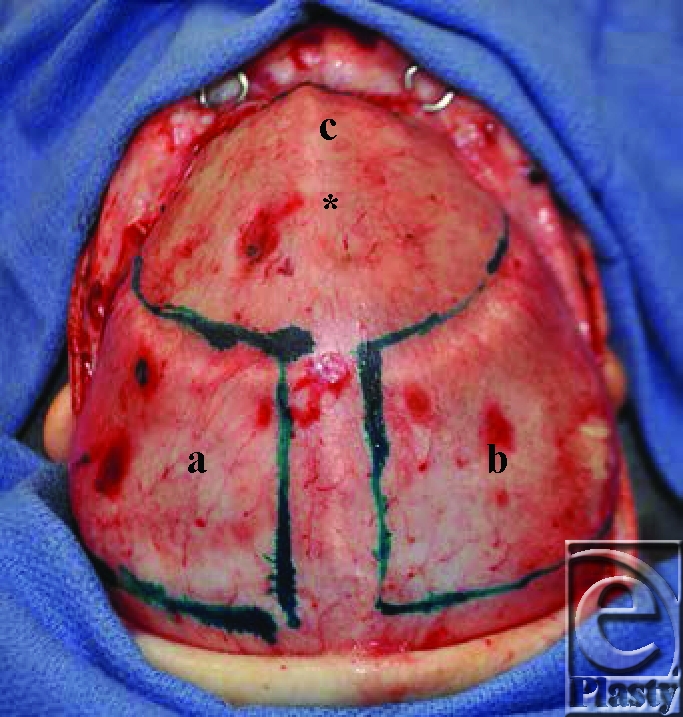
Bilateral parieto-temporal bone grafts (*a* and *b*) marked out with Brilliant Green. The superior extent of the frontal bone graft (*c*) is just visible. The fusion deformity of the metopic suture is visible in the midline (*).

**Figure 3:**
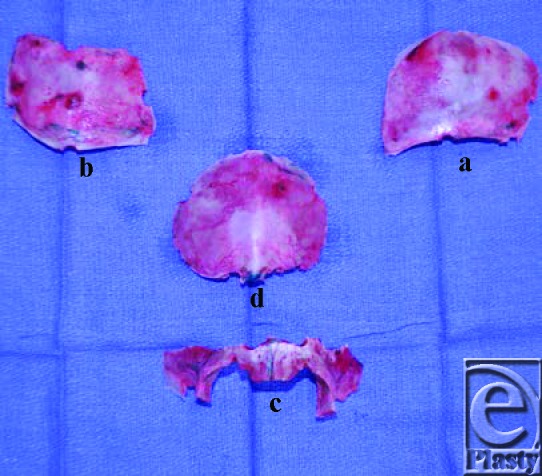
Fully mobilized bone grafts (*a*, *b*, and *c*) with the remnants of the patient's preexisting forehead (*d*), which was used as the source of bone grafts.

Brilliant Green was then used to mark out the borders of a frontal bone graft and bilateral parieto-temporal bone grafts, leaving a ridge of bone superior to the sagittal sinus intact (see Fig 2). The dura was then dissected bilaterally from the orbital roofs down to the sphenoid wings and medially until the crista gali was identified. The dissection was extended further to allow for fronto-orbital osteotomies in the superior roof of the orbit, which were subsequently extended to the lateral orbital wall. This line was then extended along the temporal bones bilaterally to provide lateral struts. Osteotomies were then performed bilaterally along the zygomatico-frontal suture lines and across the naso-frontal suture line. This allowed for full mobilization of the fronto-orbital region, which was then taken away from the operative field for construction of the new anterior cranial vault (see Fig 3).

**Figure 4:**
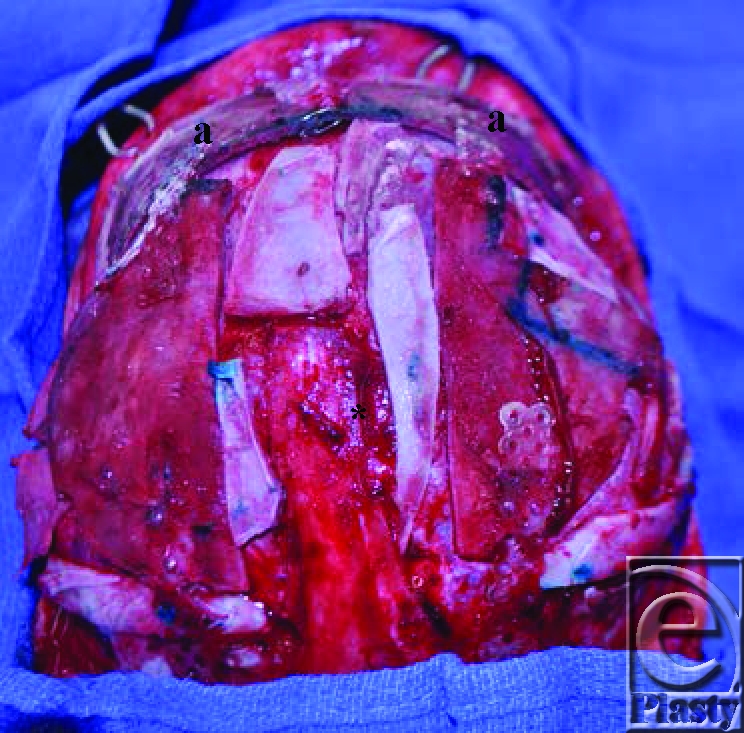
The 2 parieto-temporal bone grafts have been joined to the restructured fronto-orbital piece. The superior aspect of this new structure is visible (*a*). The remaining bone fragments have been interspersed across the parieto-temporal bony defects to form a new cranial vault.

The fronto-orbital segments were then outfractured and a bone graft was placed as a strut in the midline to prevent relapse of the orbital angulation. Next, the 2 temporal bone pieces were approximated and cut to form a new forehead piece. This was then secured to the new fronto-orbital piece, to make a complete new fronto-orbital and forehead piece. The temporal bone extensions were then infractured and secured to the remaining temporal bone using bone graft struts to maintain the advancement of the fronto-orbital bandeau. The advancement was further maintained by the use of bilateral bone graft struts between the superior orbits. Finally, the remaining original frontal bone was cut to cover the temporal defects and smaller pieces of bone graft were used to cover the remaining cranial defects (Fig 4).

The patient made an excellent postoperative recovery and was discharged 5 days after the surgery. He was reviewed in clinic 2 weeks later demonstrating a much-reduced frontal angulation and an improvement of the appearance of the cranial vault.

## Figures and Tables

**Figures 1a and 1b:**
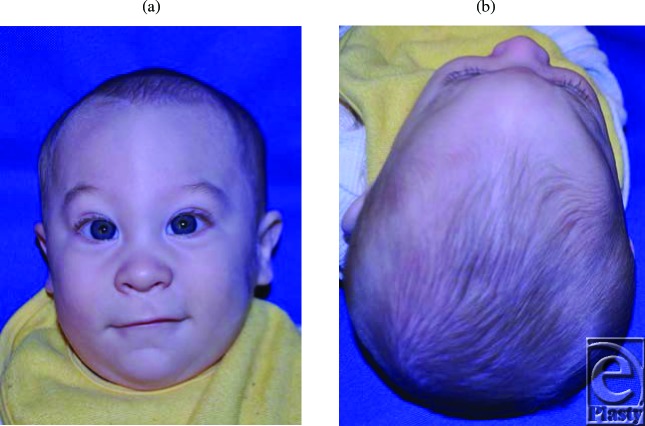
Presurgical appearance of patient showing the visible extent of the trigonocephaly.

**Figure 5:**
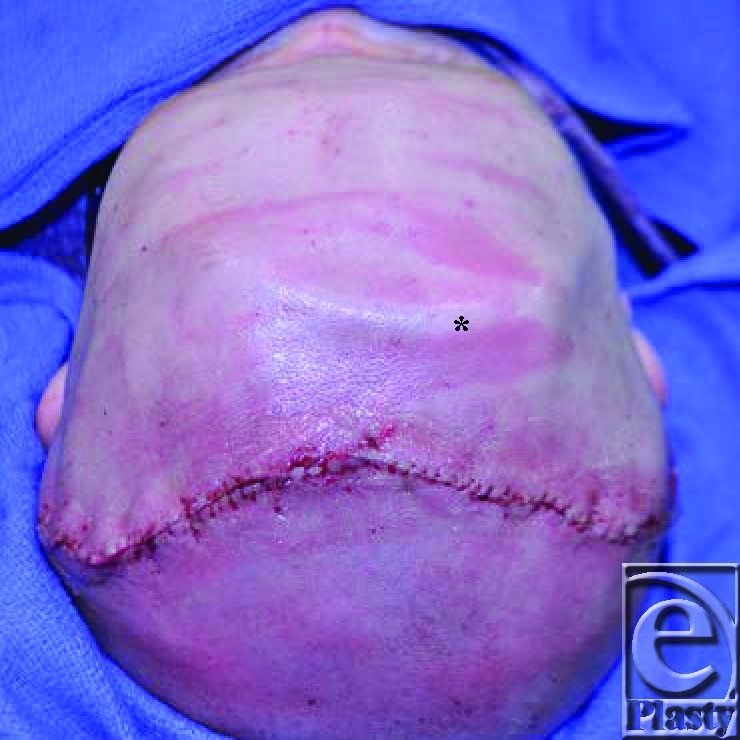
The initial postsurgical appearance showing a markedly reduced angulation of the frontal region. A subcutaneous drain is visible across the superior aspect of the cranial vault (*).

**Figures 6a and 6b:**
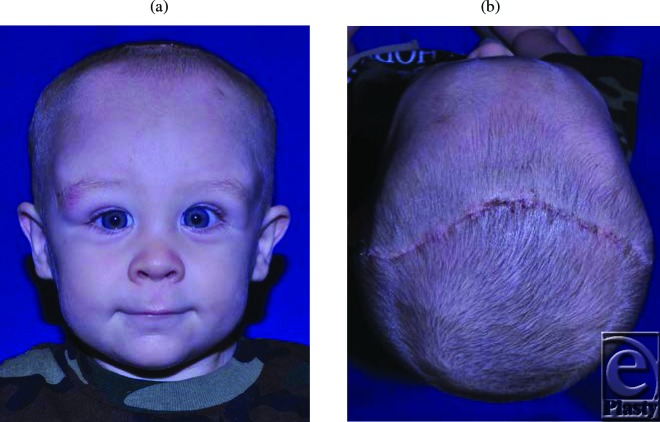
Appearance of the patient in clinic 2 weeks after surgery.
